# A Location-Based Interactive Model of Internet of Things and Cloud (IoT-Cloud) for Mobile Cloud Computing Applications †

**DOI:** 10.3390/s17030489

**Published:** 2017-03-01

**Authors:** Thanh Dinh, Younghan Kim, Hyukjoon Lee

**Affiliations:** 1 School of Electronic Engineering, Soongsil University, Seoul 06978, Korea; thanhdcn@dcn.ssu.ac.kr; 2 Department of Computer Engineering, Kwangwoon University, Seoul 01897, Korea; hlee@kw.ac.kr

**Keywords:** location interactive model, sensor cloud, IoT cloud, sensing as a service, multiple applications

## Abstract

This paper presents a location-based interactive model of Internet of Things (IoT) and cloud integration (IoT-cloud) for mobile cloud computing applications, in comparison with the periodic sensing model. In the latter, sensing collections are performed without awareness of sensing demands. Sensors are required to report their sensing data periodically regardless of whether or not there are demands for their sensing services. This leads to unnecessary energy loss due to redundant transmission. In the proposed model, IoT-cloud provides sensing services on demand based on interest and location of mobile users. By taking advantages of the cloud as a coordinator, sensing scheduling of sensors is controlled by the cloud, which knows when and where mobile users request for sensing services. Therefore, when there is no demand, sensors are put into an inactive mode to save energy. Through extensive analysis and experimental results, we show that the location-based model achieves a significant improvement in terms of network lifetime compared to the periodic model.

## 1. Introduction

Recently, the integration between Internet of Things (IoT) with the cloud (IoT-Cloud or sensor-cloud) has received significant interest from both academia and industry [1,2,3,4]. The integration is motivated by taking advantages of powerful processing and storage abilities of cloud computing for sensing data. By enabling such an integration, sensing-as-a-service (SSaaS) enables the cloud to provide sensing data to multiple applications at the same time. In addition, constrained sensor nodes can transfer processing tasks to the cloud to save energy .

Some initial studies have been conducted toward detailed design for IoT-cloud as discussed in detail in the next section. For example, in [1,2,5,6,7], the authors present architectural design for IoT-cloud. Sensor virtualization and pricing model are discussed in [8,9], while the studies [10,11,12] propose various approaches to optimize data delivery from physical wireless sensor networks to the IoT-cloud.

Although the above studies discuss how to integrate sensors with the cloud and how to distribute sensing data efficiently, few works are investigating how the sensor–cloud integration can help improve energy efficiency for resource constrained sensor nodes. In the previous work [3], we design a framework to enable the IoT-cloud to minimize the number of requests sent to shared physical sensors in the scenarios of multiple applications so that energy consumption of sensors are optimized while their sensing services still meet requirements of applications. Although some methods like request aggregation [3] or data caching [12] may help reduce data transmission, sensors are still required to transmit their data regularly. For example, in the conventional periodic sensing data collection model [3,13], sensors are required to report their sensing data periodically regardless of whether or not there are demands for their sensing services. Such a continuous sensing report without awareness of sensing demands leads to unnecessary energy loss due to redundant transmission.

In many mobile cloud computing applications such as networked robots, vehicular safety, e-health, and personal cyber-physical systems, location of mobile users are normally known by the cloud (i.e., by tracking using GPS or various location tracking schemes [14,15,16]). Therefore, when the mobile users request for sensing services, the cloud can know their location of interest. As a result, the cloud has knowledge for which location sensing services are required and in which locations sensing services are not required. For such a promising application field, the location-based on-demand interactive model between IoT and cloud can be applied, and thus need to be modeled.

This paper presents a location-based interactive model for IoT-cloud in which sensing data collection of sensors is triggered only on-demand based on mobile users’ location of interest. The model enables on-demand interaction between cloud and IoT, and exploits the cloud’s capabilities as a coordinator to save energy for resource-constrained sensors through controlling scheduling of sensors. The on-demand sensing services controlled by the cloud offer three main benefits: (1) sensors only work when needed; (2) sensing data is gathered on demand based on applications’ interest, so data redundant is reduced; and (3) sensing service quality (i.e., latency, sensing interval) can be set on-demand based on requirements of application users. We provide a comprehensive analysis of the model in comparison with the periodic sensing model under various parameters to explore when the location-based model performs better than the periodic model.

In summary, this paper makes the following contributions:

- We model a location-based interactive approach for IoT-cloud to served mobile cloud computing applications;
- We present an on-demand scheduling scheme for WSNs on the top of the model. In the scheme, the cloud plays a role as a controller that schedules sensing operations of WSNs based on mobile users’ location on demand;
- Through comprehensive analysis and experiments, we show that the location-based model achieves a significant improvement in terms of energy efficiency and network lifetime compared to the periodic sensing model.

The rest of this paper is organized as follows. Section 2 discusses related works. Section 3 presents the location-based model. Section 4 describes analysis and shows experimental results. Finally, Section 5 concludes the paper.

## 2. **Related Work**

IoT-cloud, in other words, the sensor-cloud, has been recently proposed as a promising approach that has been receiving great interest [1,2,3,5,6,7] from researchers. Although there are several main studies discussing architecture designs of IoT-cloud, their basic design is quite similar. In the IoT-cloud, physical sensors are virtualized into virtual sensors on the cloud. Physical sensor nodes are responsible for sensing and forward their sensing data to the cloud. The IoT-cloud provides sensing services directly to multiple users/applications through virtual sensors. Users/ applications request for sensing services on demand from the IoT-cloud. A number of initial studies have been conducted toward detailed design for the IoT-cloud.

In [5], Madria et al. propose an architecture for the sensor-cloud based on virtual sensors that are built on top of physical sensors. The purposes are for enhancing sensor management capability and enabling sensing data shared across multiple applications. In [2], Fazio et al. propose two models for IoT-cloud including a data-centric model and a device-centric model. In the former, the IoT-cloud offers only data to its users without exposing lower physical networks. In this model, the cloud collects physical sensing information and structures them using a uniform format to deliver to end users. In the latter, the IoT-cloud allows its users to customize virtual sensing infrastructure based on their purposes.

To optimize sensing data delivery from physical wireless sensor networks to the IoT-cloud, several studies have been investigating the issue. Misra et al. [10] design a framework using a zero-sum model for sensors to select an optimal gateway to forward their data to the cloud. The gateway selection model is mainly built based on the available bandwidth. Chatterjee et al. [11] discuss an optimal decision rule to select intermediate nodes for sensors to forward sensing data to the cloud. The decision rule is made based on different factors including Euclidean distance and energy budget. In another work [12], the same authors propose using internal and external caching techniques to save energy.

For operations of the IoT-cloud, an efficient composition method for virtual sensors is required. In [8], the authors propose two different approaches to efficiently virtualize physical sensors into virtual sensors. An optimal pricing model for the IoT-cloud is also introduced in [9]. According to the pricing model, the price of a sensing service is proportional to a number of resources it consumes. As sensors are resource constrained devices, efficient usage of sensor resources is very important to make sensing services competitive.

To further optimize resource consumptions of sensors, this paper discusses a location-based interactive model for the IoT-cloud, which enables on-demand interaction between cloud and WSNs-based location of mobile users, and exploits cloud capabilities to save energy for sensor nodes by on-demand scheduling.

## 3. The Location-Based Interactive Model

**Figure 1:**
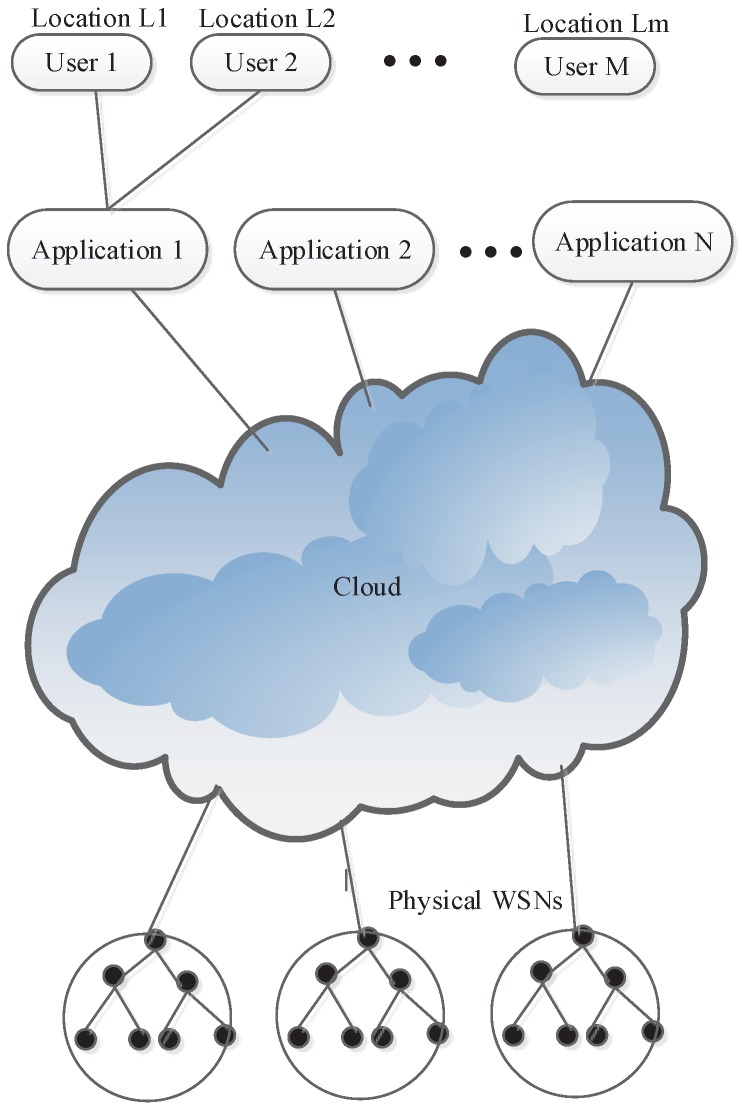
Location based IoT–cloud integration.

In this section, we describe the interactive model. First, we define main entities and functions used in the model, as shown in Figure 1.

### 3.1. Entities

**Physical Wireless Sensor Networks:** A wireless sensor network consists of physical sensor nodes. Each sensor node is characterized by the following properties: ID, type $i_{\tau }$, location *L*, and the state.

**Definition** **1.** Each physical sensor node i associates with a type $i_{\tau }$ that describes its sensor type , with $i_{\tau }\in \tau =\left\{\tau_{1},\tau_{2},\ldots ,\tau_{N}\right\}$, where τ is a set of N registered sensor types.

**Definition** **2.** After deployment, a physical sensor node registers its location with the cloud. Location $L_{i}=\left\{x_{i},y_{i}\right\}$ of a sensor node i describes the latitude and longitude of the node.

**Definition** **3.** During the lifetime, a sensor node may be in an active state (denoted by 1) or inactive state (denoted by 0). The state of a node i is denoted by $i_{\varsigma }$.

By our definition, a physical sensor *i* is modeled as follows:

$$i=\left(i_{ID},i_{\tau },i_{L},i_{\varsigma }\right),i_{\tau }\in \tau ,i_{\theta }\in \Theta .$$

**Cloud:** A cloud *c* is characterized by the following properties: ID, resources, QoS, and price. A cloud c may provide sensing service for a set of *τ* sensor types from Θ WSN owners. Note that this paper does not consider selective model for clouds, so we do not cover properties of a cloud in detail. In the IoT-cloud, sensors are virtualized into virtual sensors. We model a function that maps a physical sensor or a set of physical sensors *ζ* to a virtual sensor or a set of virtual sensors *γ* as follows:

(1) $$f_{phy->vir}\left(\zeta \right)=\gamma .$$

In our previous design [3], virtual sensors are managed by the virtual sensor manager and mapped to physical sensors by the physical sensor manager. Note that with a huge storage capability, a cloud can also act as a sensor database to provide historical sensor data services upon the demand of applications. Such a historical sensor data service can be provided directly by the sensor cloud to applications and can be built simply as a web service application. In this work, we focus on the interactive model between WSNs and the cloud, and historical sensor data services are out of the scope of this paper.

**Application:** Application *α* is characterized by the following properties: ID, a set of sensor data of interest, the region of interest, and QoS requirements (i.e., delay, sensing interval).

**Definition** **4.** An IoT-cloud application α may be interested in a set of sensor data types $\alpha_{SI}$ for its operations. These data may be requested by mobile users based on the user’s location. We later provide a function to map $\alpha_{SI}$ of an application to τ of a cloud.

**Definition** **5.** An application α is deployed to work in a limited region, called a region of interest $\alpha_{RI}=L_{1},L_{2},L_{3},L_{4}$. The region of interest consists of locations of four points that bound the region.

**Definition** **6.** Each application α may have different QoS requirements $\alpha_{QoS}$ for sensing data such as delay or sensing interval. Based on these requirements, an application may find an appropriate cloud service provider. Note that the price is normally proportional to the sensing data quality.

As a result, an application *α* is modeled as follows:

$$\alpha =(\alpha_{ID},\alpha_{SI},\alpha_{RI},\alpha_{QoS}).$$

**Mobile User:** A mobile user *μ* is characterized by the following properties: ID (i.e., device ID), application IDs $\mu_{AppIDs}$ , current location $L_{\mu }^{current}$. Note that a mobile user may use one or multiple applications. A mobile user is modeled as follows:

$$\mu =(\mu_{ID},\mu_{AppIDs},\mu_{L}^{current}).$$

We highlight that in one sensing area, there may be several mobile users who need the same sensing information.

### 3.2. Mapping Functions

We model a set of functions that used to integrate the IoT and cloud based on the location of mobile users.

*Function 1:* When an application *α* is registered with an IoT cloud, a function $f(\alpha_{SI})$ to map sensors of interest of the application to a set of actual sensor types $\tau_{\alpha }^{*}\subset \tau$ within its region of interest $\alpha_{RI}$ is modeled as follows:

(2) $$f_{1}\left(\alpha_{SI}\right)=\tau_{\alpha }^{*}=\left(\tau_{j}:\tau_{j}\in \tau \right).$$

*Function 2:* The IoT-cloud then allocates a set of virtual sensors with the same types of $\tau_{\alpha }^{*}$ that are responsible for providing sensing data of the types of $\tau_{\alpha }^{*}$ in $\alpha_{RI}$. An allocation function is defined as follows:

(3) $$f_{2}\left(\alpha_{RI},\tau_{\alpha }^{*}\right)=\gamma_{\alpha }^{*}\left(\gamma_{j}:\gamma_{j->type}\in \tau_{\alpha }^{*}and\gamma_{j->location}\in \alpha_{RI}\right).$$

These virtual sensors are assigned to provide sensing services for the the application *α*.

Sensing services in our IoT-cloud paradigm for mobile computing applications are provided based on the location of mobile users. Based on the location of mobile users, specific virtual sensors and physical sensors will be assigned on-demand. Assume a user *μ* is using an application *α* provided by the cloud. The application sends a request to the cloud for sensing data. The cloud first checks whether or not the current location of the mobile user *μ* is within the registered location of interest of the corresponding application $\alpha_{RI}$.

*Function 3:* If $\mu_{L}^{current}\in \alpha_{RI}$, a specific subset of virtual sensors that are responsible for that area will be determined to serve the user:

(4) $$f_{3}\left(\mu_{RI},\gamma_{\alpha }^{*}\right)=\gamma_{\mu }^{*}\left(\gamma_{\mu }^{*}\in \gamma_{\alpha }^{*}\&\gamma_{j->location}\in \mu_{RI}\right),$$

where $\mu_{RI}$ is the region of interest of the user *μ* with a radius of *R* around his location. The specific range *R* around the current location of *μ* depends on applications, which is not covered in this paper.

The cloud can reversely map the set of virtual sensors $\gamma_{\mu }^{*}$ to a set of corresponding physical sensors $\zeta_{\mu }$. We model the reserved mapping function as follows:

(5) $$f_{vir->phy}\left(\gamma \right)=\zeta =f_{phy->vir}^{-1}\left(\zeta \right).$$

We now discuss two interactive models for physical WSNs with the cloud. In the first model, the sensor periodically reports sensing data to the cloud. In the second model, sensors are scheduled to report sensing data on-demand based on the location of mobile users.

*Function 1:* When an application *α* is registered with an IoT cloud, a function $f(\alpha_{SI})$ to map sensors of interest of the application to a set of actual sensor types $\tau_{\alpha }^{*}\subset \tau$ within its region of interest $\alpha_{RI}$ is modeled as follows:

(6) $$f_{1}\left(\alpha_{SI}\right)=\tau_{\alpha }^{*}=\left(\tau_{j}:\tau_{j}\in \tau \right).$$

*Function 2:* The IoT-cloud then allocates a set of virtual sensors with the same types of $\tau_{\alpha }^{*}$, which are responsible to provide sensing data of the types of $\tau_{\alpha }^{*}$ in $\alpha_{RI}$. An allocation function is defined as follows:

(7) $$f_{2}\left(\alpha_{RI},\tau_{\alpha }^{*}\right)=\gamma_{\alpha }^{*}\left(\gamma_{j}:\gamma_{j->type}\in \tau_{\alpha }^{*}and\gamma_{j->location}\in \alpha_{RI}\right).$$

These virtual sensors are assigned to provide sensing services for the application *α*.

Sensing services in our IoT-cloud paradigm for mobile computing applications are provided based on the location of mobile users. Based on location of mobile users, specific virtual sensors and physical sensors will be assigned on-demand. Assume a user *μ* is using an application *α* provided by the cloud. The application sends a request to the cloud for sensing data. The cloud first checks whether or not the current location of the mobile user *μ* is within the registered location of interest of the corresponding application $\alpha_{RI}$.

*Function 3:* If $\mu_{L}^{current}\in \alpha_{RI}$, a specific subset of virtual sensors that are responsible for that area will be determined to serve the user:

(8) $$f_{3}\left(\mu_{RI},\gamma_{\alpha }^{*}\right)=\gamma_{\mu }^{*}\left(\gamma_{\mu }^{*}\in \gamma_{\alpha }^{*}\&\gamma_{j->location}\in \mu_{RI}\right),$$

where $\mu_{RI}$ is the region of interest of the user *μ* with radius of *R* around his location. The specific range *R* around the current location of *μ* depends on applications, which is not covered in this paper.

The cloud can reversely map the set of virtual sensors $\gamma_{\mu }^{*}$ to a set of corresponding physical sensors $\zeta_{\mu }$. We model the reserved mapping function as follows:

(9) $$f_{vir->phy}\left(\gamma \right)=\zeta =f_{phy->vir}^{-1}\left(\zeta \right).$$

We now discuss two interactive models for physical WSNs with the cloud. In the first model, the sensor periodically reports sensing data to the cloud. In the second model, sensors are scheduled to report sensing data on-demand based on the location of mobile users.

### 3.3. Periodic Sensing Model

In the current IoT-cloud model [3], sensors periodically report sensing data to the cloud without awareness of location and demand of mobile users. The advantage of this model is that sensing data can be updated to the cloud periodically so that sensing data are highly available to users.

However, we argue that this approach has the following limitations: (1) sensors report sensing data periodically without knowledge about demand of users, so a high volume of data may be redundant; (2) waste storage in cloud; (3) high energy consumption required by sensors; and (4) sensing data are collected with a predefined fixed QoS metrics only (i.e., delay, sensing rate). The type of model may be suitable for applications having fixed QoS requirements only while in practical applications and users may have different requirements. In many applications, sensing data are requested on demand in the context of users. When there is no demand from users, continuous sensing report is obviously unnecessary and inefficient for resource-constrained sensors.

### 3.4. Location-Based On-Demand Sensing Model

Based on the proposed model above, we propose a location-based on-demand sensing model to enable the cloud to interact with WSNs on demand based on mobile users’ location. In particular, this model schedules a sensing data report of sensors based on demand and location of mobile users. When there is no request from mobile users, inactive transmission mode is set to sensors to save energy. When there is a mobile user request from a specific area, only sensors in the area belonging to the user’s region of interest are activated to provide sensing services. As sensors are activated on demand, specific requirements of applications and users can be triggered.

The model exploits the cloud’s capability to trigger sensing in sensor nodes on demand. We assume that in mobile computing applications, the cloud has the ability to track location of users and knows when a user needs sensing data as well as at which location (i.e., by tracking using GPS or various location tracking schemes [14,15,16]), we propose to use the cloud as a coordinator to control scheduling of physical sensor nodes on demand. In particular, based on requirements of applications and current location of a mobile user, the cloud makes a scheduling request to a sensor network near the user to serve. The detail of the on-demand location-based scheduling scheme (Algorithm 1) is presented below.

**Algorithm 1** On demand location-based scheduling scheme for WSNs**Step 1:** cloud c−> current location of mobile user *μ* **Step 2:** cloud c−> allocates a set of virtual sensors and reversely map to a set of physical sensor nodes $\xi_{\mu_{L}^{current}}$ which matches with interest of the application and the user, using the above functions **Step 3:** cloud c−> makes a schedule for the sensor nodes based on current location and requirements of the mobile user **Step 5:** cloud c−> sends a scheduling request to a corresponding base station BS **Step 6:**
BS−> broadcasts the scheduling request to corresponding sensors. **Step 7:** nodes that receive the scheduling request set their own schedule based on the request. **Step 8:** when the user moves out of the area, cloud *c* sends a request to cancel the scheduling request for the set of sensors.

In our algorithm, if a node is set to be active, its parent nodes on the route to the cloud through the sink must be active too to enable the node forward sensing data. It does mean that the scheduling algorithm above has to maintain a connected graph of active sensors and the sink. We assume that locations of sensors are known and managed by the cloud. Based on location information, request packets can be forwarded from the sink to sensors efficiently using region and location-based routing protocols [17,18,19].

#### 3.4.1. Selective Nodes for Transmission

In wireless sensor networks, sensors are normally deployed densely and some nodes may obtain similar information. If the cloud requests all nodes within the region of interest to report sensing data, a great amount of data may be redundant. In our model, homogeneous virtual sensors from the same geographic region with similar sensing patterns are grouped into one cluster (i.e., corresponding to the same cluster for physical nodes) by the cloud. The similarity depends on the requirement of applications. We assume applications allow a sensing data error of err. The cloud then groups sensors whose sensing values’ difference is lower than or equal to err, into a cluster. For example, two temperature sensors having similar sensing patterns with the difference of temperature values of 0.2 ${}^{\circ }$C can be grouped into one cluster if the requirement of applications for err is greater than 0.2 ${}^{\circ }$C. In this case, only one of the two nodes is required to report sensing data at a time to eliminate data redundancy. Sensors within a cluster produce similar data, so if they all report their data, a high redundancy will occur. Therefore, in each round, the cloud selects only one virtual sensor (i.e., corresponding to one physical node) within a cluster to serve mobile users to eliminate redundancy. Other sensors of the same cluster can sleep to save energy. For energy balancing, each node in a cluster is responsible as the active node for reporting data in one round if there are sensing demands. The role of active node is switched after each round (i.e., the length of one round is normally quite long, and equals a number of request intervals). For example, we assume that there is a cluster of four nearby temperature sensors that produce similar sensing data. It is inefficient when all four of the sensors are required to be active and transmit their data to the sensor cloud. In our model, only one of the four nodes is required to be active and report its sensing data to the cloud at a time when there are demands. The other three nodes can sleep to save energy. For energy balancing, in each round, a sensor with the highest residual energy among the four nodes is selected as the active node.

Note that all operations are executed on the cloud, so no complexity is put into resource-constrained sensors. We model the cluster discovery of a virtual sensor *ν* using network community discovery theory [20]. According to [20], we can define constraints so that the difference between sensing data *ν* and other virtual sensors is minimized. Given a set of *n* virtual sensors N=1,2,…,n within a geographical region, a virtual sensor *ν*, determines a cluster $C_{\nu }$ and a regression function *ω* so that the difference, expressed through the loss function $\Phi \left(\nu ,\omega \right)=Loss(d_{\nu },\omega \left(d_{C_{\nu }}\right))$, where *d* are sensing values, is minimized.

We assume that *ω* is linear and Φ is mean square error (MSE). For lowering the difference, we need to find a group of node $C_{\nu }\;\nu$ so that a decision *ζ* is minimized:

(10) $$E\left[\Phi \left(D,\zeta \right)\right]=E\left[(D_{\nu }-\zeta^{T}D_{C_{\nu }}{)}^{2}\right].$$

In the formula, $D_{x}$ is a random vector consisting of $D_{\nu }$. The cluster discovery relies on a given historical data set consisting of *h* samples $D=[d_{1}d_{2}\ldots d_{m}]$. As correlations of nodes may change over time, the cluster discovery is updated periodically every round. We can apply a heuristic solution to discover a decision $\zeta_{\nu }$ based on the given data set so that it can obtain the number of zero entries of *ζ* as high as possible. In other words, we should look for a sparse decision $\zeta_{\nu }$ that minimizes the mean square error (MSE) and the $L_{1}$ norm. This is a typical Least Absolute Shrinkage and Selection Operator (LASSO) problem [21], which can be solved easily by using a LASSO parameter *ρ* as follows:

(11) $$minimize\{\rho ∥\zeta_{k}{∥}_{1}+1/2∥d_{\left[-\nu \right]}\zeta_{\nu }-d_{\nu }{∥}_{2}^{2}\}.$$

As a result, the cluster $C_{\nu }$ is discovered as a set of virtual nodes with non-zero entries of $\zeta_{\nu }$. The parameter *ρ* is used to control the expected error as well as the sparsity of *ζ*—in other words, the size of $C_{\nu }$. To meet the requirement of applications and to find the largest cluster, *ρ* is actually the allowed error err.

#### 3.4.2. Multiple Mobile Users within a Geographical Region

The IoT cloud tracks all mobile users who are currently using sensing services in a geographical region. When the cloud receives a request from a new user, it first checks if there are available data from the region of interest requested by other users. If there are available data meeting requirements of new users, the cloud reuses the data for later coming users.

## 4. Performance Evaluation

This section presents analysis and evaluation for the model. We compare the performance of the location-based model with traditional periodic sensing WSNs. In our evaluation, we focus on energy efficiency and network lifetime of WSNs. In both cases, we assume that WSNs operate in duty cycle mode using a low power listing (LPL) protocol [13] and the base station is always on.

### 4.1. Performance Analysis

A sensor node mainly consumes energy for transmitting (tx), receiving (rx), listening (lx), sensing, and computing. The analysis for energy consumption is as follows:

$$E=E_{lx}+E_{tx}+E_{rx}+E_{sensing}+E_{computing}.$$

We translate the energy consumption metric to average duty cycle DC as an indicator for energy efficiency. The reason is that duty cycle is mostly hardware independent and easier to reproduce results:

$$DC=DC_{lx}+DC_{tx}+DC_{rx}+DC_{sensing}+DC_{computing},$$

where $DC_{lx},DC_{tx},DC_{rx},DC_{sensing},and\;DC_{computing}$ are ratio of amount of time per a second a sensor node spends for listening, transmitting, receiving, sensing, and computing. The detailed medium access control (MAC) configuration and traffic model for sensors are presented in our previous work [13].

To keep the analysis tractable, we use a concentric circular ring network (CCR) model [13] with the sink as the center for the network deployment in each region. The sink node interconnects sensors to the cloud. Nodes communicate with each other based on a unit disk graph model. Sensors are uniformly deployed to achieve the same density with D + 1 nodes per a unit disk (i.e., D neighbors per node).

Each CCR *h* consists of sensor nodes with the same minimum hop count *h* to the sink. The number of sensors in the first CCR is equal to the number of sink’s neighbors. Based on that, we calculate the number of nodes $N_{h}$ on the CCR $h_{th}$ as follows:

(12) $$N_{h}=\left\{\begin{array}{cc} 1, & \;\mathrm{if}\;h=0,\\ Dh^{2}-D{\left(h-1\right)}^{2}=D\left(2h-1\right), & \;\mathrm{otherwise}. \end{array}\right.$$

Nodes on the hop (h+1) are children of nodes on the hop *h*. As each node determines one parent node, we can calculate the average number of child nodes $|C_{h}|$ of a node in hop *h*:

(13) $$|C_{h}|=\left\{\begin{array}{cc} 0, & \;\mathrm{if}\;h=h_{max},\\ D, & \;\mathrm{if}\;h=0,\\ N_{h+1}/N_{h}=\left(2h+1\right)/\left(2h-1\right), & \;\mathrm{otherwise}, \end{array}\right.$$

where $h_{max}$ indicates leave nodes.

We call $F_{self}$ as traffic generated by a node, and $F_{in}\left(h\right)$ as the average input traffic rate of a node at hop *h*. The average output traffic rate of the node $F_{out}\left(h\right)$ can be computed by adding $F_{self}$ and $F_{in}\left(h\right)$:

(14) $$F_{out}\left(h\right)=\left\{\begin{array}{cc} F_{self}, & \;\mathrm{if}\;h=h_{max,}\\ F_{in}\left(h\right)+F_{self}, & \;\mathrm{otherwise}. \end{array}\right.$$

The input traffic rate at a node in CCR *h* is the total of output rate at its input links produced by its child nodes. Therefore, we can calculate the output traffic rate of a node $F_{out}\left(h\right)$ at hope *h* as the cumulative traffic of nodes from leaves to nodes at level h+1 on its route and its self-generated traffic as follows:

(15) $$F_{out}\left(h\right)=F_{self}\left(h_{max}^{2}-h^{2}+2h-1\right)/\left(2h-1\right).$$

It does mean that a node at hop *h* is responsible for carrying out traffic for $C=\left(h_{max}^{2}-h^{2}+2h-1\right)/\left(2h-1\right)$ nodes under its route.

Duplicate sensing data depends on the deployment of sensors. We assume that the average size of a cluster is *X* (i.e., each node has similar sensing pattern with (X−1) other nodes). Each node has an initial energy of $E_{0}$. Periodic sensing interval of a node used in the periodic sensing model is $I_{periodic}$ while we denote $I_{request}$ as the average request interval of mobile users for a specific node. The main difference of energy consumption between the two models comes from packet transmitting and receiving. To keep the analysis tractable, we mainly focus on these two factors in calculating the energy consumption of sensors in the two models for comparison. Note that, for a fair comparison, we do not assume any type of aggregation applied for both of the models.

Energy consumption rate of a node in the periodic model is:

(16) $$E_{periodic}=\frac{1}{I_{periodic}}\left(C+1\right)T_{x}+CR_{x},$$

where $T_{x}$ and $R_{x}$ are energy consumption rate for each transmitting and receiving time.

The estimated network lifetime *L* for a node using the periodic model is

(17) $$L=\frac{E_{0}}{E_{periodic}}=\frac{E_{0}\times I_{periodic}}{\left(C+1\right)T_{x}+CR_{x}}.$$

Energy consumption rate of a node in the location-based model is:

(18) $$E_{location}=\frac{1}{I_{request}}\frac{1}{X}\left\{\left(C+1\right)T_{x}+CR_{x}+E_{s}\right\},$$

where $E_{s}$ is the scheduling cost if the node has to relay scheduling requests. This cost also depends on $I_{request}$.

The estimated network lifetime *L* for a node using the location-based model is

(19) $$L=\frac{E_{0}}{E_{location}}=\frac{E_{0}\times I_{request}\times \left(X\right)}{\left(C+1\right)T_{x}+CR_{x}+E_{s}}.$$

The packet delivery latency *L* regarding to LPL protocol [13] is analyzed as follows:

(20) $$L_{h}=\left(h-1\right)\left(I_{W}/2+T_{pkt}\right)+T_{pkt},$$

where *h* is the number of hops to the sink, $I_{W}$ is sleep interval, and $T_{pkt}$ is the time period to transmit a data packet. The detailed analysis and results for latency can be found in [13]. We calculate the above parameters based on default values of the TOSSIM radio model for CC2420 used in [13].

Each model has a different policy for periodic wakeup, sensing, and packet transmitting as follows.

**The Periodic Model:** In the periodic model, a node periodically wakes up, performs sensing, processing, and transmits sensing packets to the cloud. In this model, WSNs may be deployed without prior knowledge of where and when a user may request sensing data. They are set beforehand with a reasonable wakeup interval $I_{W}$ and sensing data report interval $I_{S}$ for the trade-off between energy efficiency and requirements of general applications (i.e., in default LPL setting, $I_{W}^{WSN}=0.5$ s is used and $I_{S}^{WSN}=10$ s is popularly used).

**The Location-Based Model:** In the location-based model, because the cloud is aware of the location of users and when sensing data is requested in a region of interest of an application, it can control when a sensor needs to provide sensing service and at which rate to meet requirements of served applications. According to state-of-the-art localization and prediction algorithms, the cloud can track and estimate the near future (i.e., a few seconds or even more) locations of mobile users. The cloud sends scheduling requests to WSNs to enable them to be available to serve mobile users when the users enter their region of interests. For example, when there is no user demand, WSNs can be set with $I_{W}^{quiet}=1$ s and $I_{S}^{quiet}=\infty$. With a longer sleep interval and without a need for data packet transmission when there are no serving requests, the model helps save energy for constrained sensor nodes. When a mobile user requests sensing data from a WSN, sensors can be scheduled to wake up more frequently (i.e., $I_{W}^{serving}=0.25$ s or even always on) and perform high sensing rate (i.e., $I_{S}^{serving}=1$ s or even lower) depending on requirements of the application, to serve mobile users. With lower sleep interval and higher sensing rate settings on demand, the model can provide a better quality of sensing information to applications and users. In summary, the main benefit of the proposed work is that based on knowledge of the cloud about where and when sensing services are required, WSNs are scheduled to (1) save as much energy as possible during quite time periods and (2) be available to serve users on demand with a high quality of information based on requirements of applications.

### 4.2. Numerical Results

In this section, we present the analysis and experimental results of the two models. Detailed parameter specification is given in Table 1. Other parameters are set to the default values of the TOSSIM simulator radio model for CC2420, inherited from our previous work [3]. To measure the duty cycle, we record changes in the radio’s states and use a counter to accumulate the time period used in each state.

**Figure 2:**
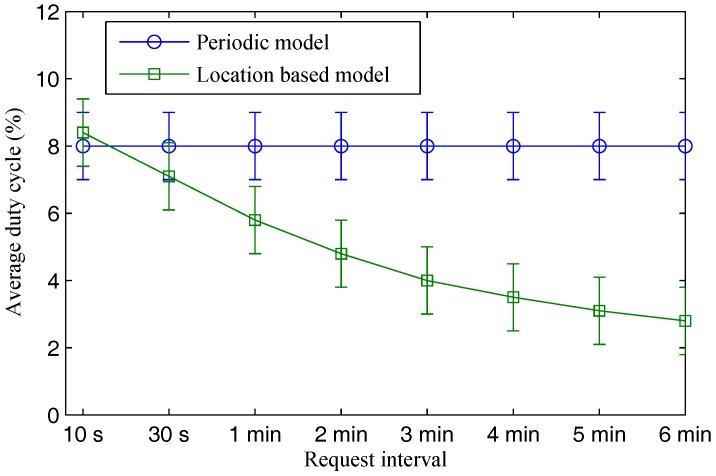
Average energy consumption under various request intervals.

Figure 2 shows average duty cycle results in various request interval values. While the duty cycle result of the location-based model highly depends on the request interval value, that of the periodic model is independent to the request interval. In the periodic model, sensors keep reporting their sensing data regardless of whether or not there are demands. On the contrary, energy consumption in the location-based model is proportional to the request frequency of mobile users. When there are high demands (i.e., short request interval), sensors have to transmit their sensing data more frequently. Therefore, higher duty cycle results are observed. If a high frequency of requests is sent (i.e., request interval of 10 s), average duty cycle of the location-based model is even higher than the periodic model (i.e., with periodic interval of 20 s). In this case, using a periodic model may be more beneficial in terms of energy consumption for sending requests. When the request interval increases, average duty cycle result of the location-based model is inversely proportional. At the request interval of 30 s, duty cycle result of the location-based model is considerably lower than that of the periodic model. After that, the average duty cycle result of the location-based model significantly drops to around 3% when the request interval is increased to 6 min, compared to 8% of the periodic model.

**Figure 3:**
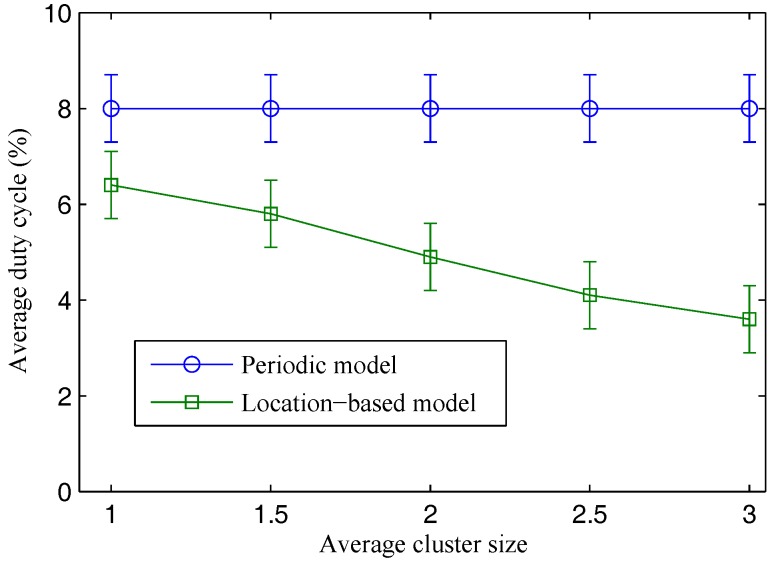
Average energy consumption under various cluster sizes.

Figure 3 presents the performance behavior of the two models under different average cluster sizes—in other words, under different correlation levels of sensing data among nodes. In the periodic model, every node periodically reports its data to the cloud without knowing there are demands or not. In the location-based model, the cloud plays a role as the controller who controls which nodes are required to report their data under specific requests of mobile users. Therefore, the cloud can reduce data redundancy by minimizing the number of nodes that produce similar sensing data required to report their data at the same time. In this way, the model can achieve more energy savings compared to the periodic model under equivalent conditions (i.e., request interval, packet size, low power listening mode, etc.). Energy efficiency of the location-based model is proportional to the average cluster size. At a cluster size of 1, the two models witness a small difference in the average duty cycle results. The energy efficiency of the location-based model is increased significantly when we increase the average cluster size from 1 to 3. This result indicates that the location-based model is more effective in cases of dense network deployment with high redundancy.

**Figure 4:**
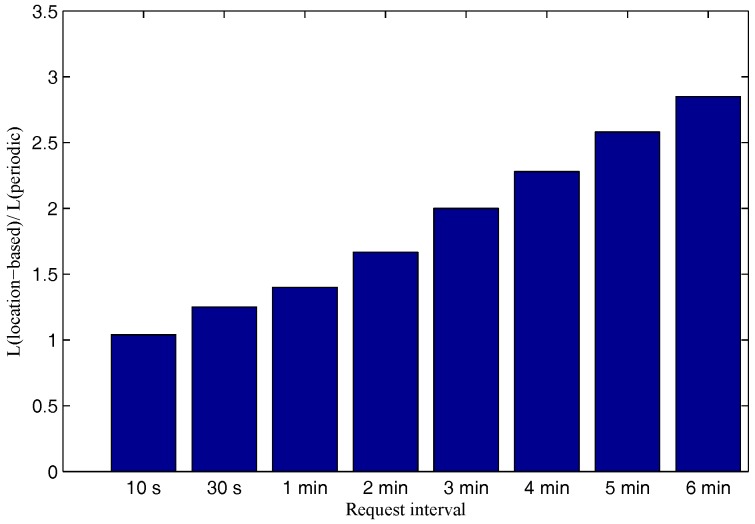
Network lifetime improvement vs. request intervals.

Figure 4 provides how much improvement in terms of the network lifetime that the location-based model achieves compared to the periodic model. Note that the network lifetime in this paper is defined as the lifetime of the network until there is at least one node out of battery. The network lifetime improvement of the location-based model compared to the periodic model is proportional to the request interval. In particular, the improvement increases from only 4% to more than two times when the request interval increases from 10 s to 6 min.

**Figure 5:**
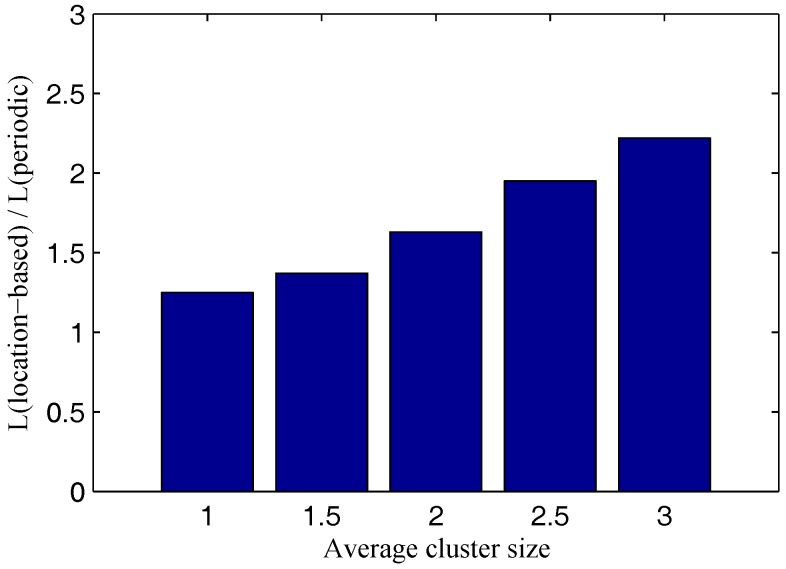
Network lifetime improvement vs. cluster sizes.

Similar to Figure 4, Figure 5 shows the network lifetime improvement of the location-based model under various cluster sizes. The stronger the data correlation among sensors in a network, the greater the network lifetime improvement that the location-based model can achieve.

**Scenarios with High Error Localization Systems:** In this paper, we assume mobile devices have GPS or use location tracking services like StarTrack. The accuracy of the current localization techniques is good enough for our model as well as similar existing location-based applications like mobility pattern monitoring of moving objects in large cities [22], continuous multi-dimensional context and activity recognition [23], criminal tracking, autonomous car navigation and obstacle avoidance [24,25]. Real-world data from the Federal Aviation Administration show that their GPS attains better than 2.168 m accuracy with a 95% confidence level [26]. Higher accuracy is attainable by using GPS in combination with augmentation systems that enable real-time positioning to within a few centimeters.

In cases of a high error localization system, the model can still ensure the region of interest of a user by compensating for the localization error into the region of sensor data collection. We conduct a simple analysis for illustration. Given a sensor network deployment area has a number of sensors. The average distance between two sensors is 8 m and the transmission range is 10 m. The average cluster size is 3. The region of interest of a user has a radius of 50 m.

We assume the error of the used localization technique is 10 m. To ensure the user’s region of interest, the model collects sensor data in a region of 60 m (i.e., 50 m + 10 m). In this case, more nodes are required to be involved in providing sensing data collection. As a result, energy efficiency of the proposed model is reduced by 12%, compared to the case of an accurate localization system. Although the improvement ratio is reduced due to a high localization error, energy efficiency of the proposed model is still significantly better than the conventional model.

## 5. Conclusions

This paper proposes a location-based sensing model for mobile cloud computing applications, in comparison to the conventional periodic sensing model. In the proposed location-based model, the IoT-cloud provides sensing services on demand based on interest and location of mobile users. Based on mobile user location tracking, the IoT-cloud plays a role as a controller, which makes schedules for physical sensor networks on-demand. In this way, resource-constrained sensors are required to report their sensing data only when there is a mobile user entering their region and requesting for sensing data. Otherwise, they are set in power saving mode to save energy. Through analysis and experimental results, we show that the proposed location-based model improves the network lifetime of wireless sensor networks significantly. 

## Figures and Tables

**Table 1:** Parameters.

Parameter	Value	Parameter	Value
Data packet length	32 bytes	Nodes	100
Carrier sensing	2.5 ms	Sensing	2 ms
$I_{W}^{WSN}$	0.5 s	$I_{S}^{WSN}$	10 s
$I_{W}^{serving}$	0.25 s	$I_{S}^{serving}$	1 s
$I_{W}^{quiet}$	1 s	$I_{S}^{quiet}$	∞
Θ	1–5	# of apps	1–5
$I_{request}$	10 s–10 min	$T_{request}$	4 s
*h*	5	# of nodes	126
*ρ*	0.5	$T_{pkt}$ /byte	0.032 ms

## References

[1] Babu S., Lakshmi A., Rao B. A study on cloud based Internet of Things: CloudIoT. Proceedings of the Global Conference on Communication Technologies (GCCT).

[2] Fazio M., Puliafito A. (2015). Cloud4sens: A cloud-based architecture for sensor controlling and monitoring. IEEE Commun. Mag..

[3] Dinh T., Kim Y. (2016). An Efficient Interactive Model for On-Demand Sensing-As-A-Services of Sensor-Cloud. Sensors.

[4] Dinh T., Kim Y., Lee H. A location-based interactive model for Internet of Things and cloud (IoT-cloud). Proceedings of the Eighth International Conference on Ubiquitous and Future Networks (ICUFN).

[5] Madria S., Kumar V., Dalvi R. (2014). Sensor Cloud: A Cloud of Virtual Sensors. IEEE Softw..

[6] Santos I., Pirmez L., Delicato F., Khan S., Zomaya A. (2015). Olympus: The Cloud of Sensors. IEEE Cloud Comput..

[7] Sheng Z., Wang H., Yin C., Hu X., Yang S., Leung V. (2015). Lightweight Management of Resource-Constrained Sensor Devices in Internet of Things. IEEE Internet Things J..

[8] Chatterjee S., Misra S. Optimal composition of a virtual sensor for efficient virtualization within sensor-cloud. Proceedings of the IEEE International Conference on Communications (ICC).

[9] Chatterjee S., Ladia R., Misra S. (2015). Dynamic Optimal Pricing for Heterogeneous Service-Oriented Architecture of Sensor-cloud Infrastructure. IEEE Trans. Serv. Comput..

[10] Misra S., Bera S., Mondal A., Tirkey R., Chao H.C., Chattopadhyay S. (2014). Optimal gateway selection in sensor-cloud framework for health monitoring. IET Wirel. Sens. Syst..

[11] Chatterjee S., Sarkar S., Misra S. Energy-efficient data transmission in sensor-cloud. Proceedings of the Applications and Innovations in Mobile Computing (AIMoC).

[12] Chatterjee S., Misra S. Dynamic and adaptive data caching mechanism for virtualization within sensor-cloud. Proceedings of the IEEE International Conference on Advanced Networks and Telecommuncations Systems (ANTS).

[13] Dinh T., Gu T., Vasilakos A., Kim Y. (2016). L-MAC: A Wake-up Time Self-learning MAC Protocol for Wireless Sensor. Comput. Netw..

[14] Bhattacharya S., Blunck H., Kjærgaard M.B., Nurmi P. (2015). Robust and Energy-Efficient Trajectory Tracking for Mobile Devices. IEEE Trans. Mob. Comput..

[15] Zhuang Z., Kim K.H., Singh J.P. Improving Energy Efficiency of Location Sensing on Smartphones. Proceedings of the 8th International Conference on Mobile Systems, Applications, and Services (MobiSys ’10).

[16] Ananthanarayanan G., Haridasan M., Mohomed I., Terry D., Thekkath C.A. StarTrack: A Framework for Enabling Track-based Applications. Proceedings of the 7th International Conference on Mobile Systems, Applications, and Services (MobiSys ’09).

[17] Lee J., Park H., Kang S., Kim K.I. (2015). Region-Based Collision Avoidance Beaconless Geographic Routing Protocol in Wireless Sensor Networks. Sensors.

[18] Saad C., Benslimane A., Champ J., Konig J.C. Ellipse Routing: A Geographic Routing Protocol for Mobile Sensor Networks with Uncertain Positions. Proceedings of the 2008 IEEE Global Telecommunications Conference.

[19] Zhang H., Shen H. (2010). Energy-Efficient Beaconless Geographic Routing in Wireless Sensor Networks. IEEE Trans. Parallel Distrib. Syst..

[20] Fortunato S. (2010). Community detection in graphs. Phys. Rep..

[21] Tibshirani R. (1996). Regression Shrinkage and Selection via the Lasso. J. R. Stat. Soc. Ser. B Methodol..

[22] Kim B., Lee S., Lee Y., Hwang I., Rhee Y., Song J. (2011). Mobiiscape: Middleware support for scalable mobility pattern monitoring of moving objects in a large-scale city. J. Syst. Softw..

[23] Bhargava P., Gramsky N., Agrawala A. SenseMe: A System for Continuous, On-device, and Multi-dimensional Context and Activity Recognition. Proceedings of the 11th International Conference on Mobile and Ubiquitous Systems: Computing, Networking and Services (MOBIQUITOUS ’14).

[24] Fernandez C., Dominguez R., Fernandez-Llorca D., Alonso J., Sotelo M.A. (2013). Autonomous Navigation and Obstacle Avoidance of a Micro-Bus. Int. J. Adv. Robot. Syst..

[25] Luettel T., Himmelsbach M., Wuensche H.J. (2012). Autonomous Ground Vehicles—Concepts and a Path to the Future. Proc. IEEE.

[26] GPS Accuracy. http://www.gps.gov/systems/gps/performance/accuracy/.

